# Advanced Neural Functional Imaging in *C. elegans* Using Lab-on-a-Chip Technology

**DOI:** 10.3390/mi15081027

**Published:** 2024-08-12

**Authors:** Youngeun Kwon, Jihye Kim, Ye Bin Son, Sol Ah Lee, Shin Sik Choi, Yongmin Cho

**Affiliations:** 1 Department of Chemical Engineering, Myongji University, Yongin 17058, Republic of Korea; kye3526@mju.ac.kr (Y.K.); wisdom9952@mju.ac.kr (J.K.); sonyebin0106@mju.ac.kr (Y.B.S.); 2 School of Chemical & Biomolecular Engineering, Georgia Institute of Technology, Atlanta, GA 30332, USA; solemail8987@gmail.com; 3 Department of Bio-Pharmaceutical Sciences, Myongji University, Yongin 17058, Republic of Korea; sschoi@mju.ac.kr; 4 The Natural Science Research Institute, Department of Food and Nutrition, Myongji University, Yongin 17058, Republic of Korea; 5 elegslab Inc., Seoul 06083, Republic of Korea

**Keywords:** microfluidics, *C. elegans*, neural functional imaging, calcium imaging

## Abstract

The ability to perceive and adapt to environmental changes is crucial for the survival of all organisms. Neural functional imaging, particularly in model organisms, such as *Caenorhabditis elegans*, provides valuable insights into how animals sense and process external cues through their nervous systems. Because of its fully mapped neural anatomy, transparent body, and genetic tractability, *C. elegans* serves as an ideal model for these studies. This review focuses on advanced methods for neural functional imaging in *C. elegans*, highlighting calcium imaging techniques, lab-on-a-chip technologies, and their applications in the study of various sensory modalities, including chemosensation, mechanosensation, thermosensation, photosensation, and magnetosensation. We discuss the benefits of these methods in terms of precision, reproducibility, and ability to study dynamic neural processes in real time, ultimately advancing our understanding of the fundamental principles of neural activity and connectivity.

## 1. Introduction

All organisms, from humans to the simplest forms of life, are profoundly attuned to environmental changes. The ability to perceive and adapt to these changes is crucial for survival as it enables organisms to navigate their surroundings, find food, and avoid danger. Understanding how organisms perceive external cues through their nervous systems requires advanced technologies, among which neural functional imaging stands out. This technology allows researchers to observe neuronal activity in real time, providing a dynamic view of how neurons communicate, respond to stimuli, and influence behavioral and cognitive processes [1]. The significance of neural imaging extends beyond basic understanding; it plays an instrumental role in unraveling the mysteries of neural development, synaptic plasticity, and neural circuitry [2]. Neural imaging is vital in the study of neurodegenerative diseases [3,4], and can help identify pathological changes in the brain before the emergence of clinical symptoms.

*Caenorhabditis elegans*, a microscopic free-moving nematode, is a powerful model organism for neural imaging for several reasons. First, its fully mapped neural anatomy, comprising 302 neurons in hermaphrodites, presents a simplified yet comprehensive system for exploring complex neural networks [5,6,7,8,9]. Second, the transparent body and deterministic neural architecture of the nematode allow direct observation of neuronal activity using noninvasive imaging techniques, enabling researchers to monitor neuronal responses in live, active specimens [10]. Third, the genetic tractability of *C. elegans*, with its well-characterized genome, allows for the precise manipulation of specific genes to study their effects on neural function and behavior, providing key insights into the genetic basis of cognitive processes and neurodegenerative diseases [11]. Finally, the short lifecycle and cost-effective and straightforward maintenance of *C. elegans* enable the generation of large isogenic populations [12,13]. This allows the observation and understanding of neuronal activity across numerous individuals, significantly reducing variability and enhancing the accuracy of findings. Collectively, these features make *C. elegans* a practical and powerful tool for gaining deep insights into the fundamental principles of neural activity and connectivity, which are applicable to more complex organisms.

Lab-on-a-chip technology has emerged as a revolutionary tool in the neural imaging field, particularly for studying neural functional imaging in model organisms, like *C. elegans,* with unprecedented capabilities. This technology integrates microfluidics and other miniaturized systems to create precise and controlled microenvironments that are essential for accurate and repeatable experiments. Lab-on-a-chip platforms facilitate the manipulation of minute quantities of fluids, the application of precise stimuli, and the real-time observation of biological events, such as neuronal activity and behaviors, within a single, compact system, all while preserving the viability and functionality of the biological specimen. Moreover, these platforms are designed for high-throughput experiments, enabling the rapid and parallel processing of numerous samples. This approach significantly accelerates data collection and analysis while also improving the consistency of results by reducing the influence of biological variability.

In this review, we discuss advanced methods for neural functional imaging in *C. elegans*, focusing on lab-on-a-chip technologies that provide innovative solutions for controlling environmental cues and delivering precise stimuli while recording neuronal activity. These microfluidic devices offer unprecedented accuracy and repeatability, enabling detailed studies of neural responses to various sensory inputs, such as chemical, mechanical, thermal, photonic, magnetic, and multimodal stimuli [14]. By employing these advanced methods, researchers can achieve high-resolution, real-time observations of neural activity, thereby gaining deeper insight into the complex dynamics of neural function and behavior in *C. elegans*.

## 2. Neuronal Function Studies in *C. elegans* with Calcium Imaging Techniques

Calcium imaging has become a fundamental tool in neuroimaging for the real-time observation of neural activity. This technique is particularly advantageous for studying the neural functions of *C. elegans*, given its transparent body and genetically encoded calcium indicators, such as the GCaMP family and FRET-based indicators (e.g., chameleon) [15,16,17,18,19,20]. Calcium imaging capitalizes on the fluorescence properties of these indicators, which exhibit changes in intensity correlated with fluctuations in calcium ion concentrations within neurons [21]. These changes are indicative of neural activity, as calcium ions play a crucial role in neuronal signaling. Numerous transgenic strains expressing these indicators in specific neurons are readily available or can be generated using well-established genetic techniques. For those seeking detailed methodologies for measuring calcium dynamics in *C. elegans*, comprehensive guides by Chung et al. are available [22], and Kerr has provided an in-depth overview of these imaging techniques in ”Imaging the Activity of Neurons and Muscles” on WormBook [23].

Achieving high-quality neural functional imaging in *C. elegans* necessitates the effective immobilization of worms within the field of view with highly controlled external stimuli, a process that presents several technical challenges. For example, a pharmacological agent such as levamisole, which is a cholinergic agonist that induces muscle paralysis, is useful for immobilizing worms during microscopic examinations without causing permanent harm, whereas physical immobilization can be achieved using veterinary-grade glue. However, determining whether pharmacological agents or organic glue are toxic to *C. elegans* and their effect on neuronal activity is challenging [24]. Additionally, the glue approach restricts the procedure to single-animal studies, results in low-throughput experiments, and does not allow animal recovery.

## 3. Advances in Lab-on-a-Chip Technology for Neural Imaging Systems in *C. elegans*

Lab-on-a-chip technologies and microfluidic devices have emerged as transformative approaches in the field of neural functional imaging in *C. elegans*, offering innovative solutions to the many limitations inherent in traditional imaging methods. One of the primary benefits of these miniaturized devices is their ability to precisely control environmental conditions. Traditional methods often struggle with the precise manipulation of external stimuli owing to limitations in environmental control and the granularity of adjustments. In contrast, microfluidic devices excel in this area, allowing researchers to finely tune a range of experimental stimuli, such as chemical gradients, temperature, and mechanical forces, which can be applied with unprecedented accuracy and repeatability [25]. A key feature of microfluidic devices is their ability to generate and maintain a laminar flow, which ensures minimal mixing of adjacent fluid streams [26]. This characteristic allows for precise control of fluid flow within the device, facilitating the creation of well-defined and stable microenvironments. These controlled conditions enable the delivery of precise stimuli, enhance the precision of neural functional imaging, and yield reliable and reproducible results [27].

Additionally, these devices are particularly well suited for manipulating small organisms, such as *C. elegans* [28,29]. The design of microfluidic channels allows for the precise positioning and orientation of multiple specimens at a time or sequentially within the device, facilitating high-quality neural functional imaging. The ability to precisely confine *C. elegans* within microchannels often eliminates the need for drug-induced immobilization because the physical constraints of the device itself can effectively restrict movement [30]. This not only preserves the physiological state of the animals but also facilitates their easy recovery after studies. Such recovery is crucial, as it allows for the subsequent tracking of the same animals across multiple assays, providing a consistent and controlled method for studying longitudinal effects.

**Figure 1:**
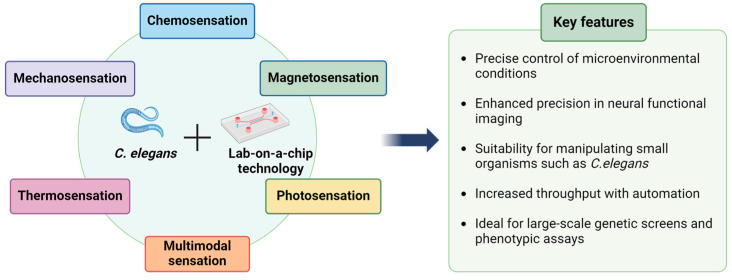
Schematic representation of the key features of lab-on-a-chip technology for neuronal functional imaging in *C. elegans.* Image elements of this figure were created using BioRender.

Finally, when integrated with automated technologies, lab-on-a-chip systems significantly enhance throughput, while reducing time and resource consumption [31,32]. This efficiency is particularly advantageous for obtaining robust results in studies on inherently noisy responses in neuronal cells. These systems are also ideal for conducting large-scale genetic screens or phenotypic assays that involve the analysis of thousands of specimens, tasks that are well suited to the rapid lifecycle and straightforward maintenance of *C. elegans* [33,34,35]. The synergy between lab-on-a-chip technologies and *C. elegans* as a model organism provides a powerful platform for advancing our understanding of neuroscience (Figure 1). This section explores advanced methods for neural functional imaging in *C. elegans*, with a particular focus on lab-on-a-chip technologies (Table 1). We delve into technologies and techniques that enable precise manipulation and observation of neural activities across various sensory modalities.

### 3.1. Chemosensation

Chemosensation is a process by which organisms detect and respond to chemical stimuli in their environment. This ability is crucial, as it helps organisms find food, avoid harmful conditions, and locate mates. Despite its small nervous system, *C. elegans* possesses remarkable chemosensory capabilities, with 20–30 chemosensory neurons capable of detecting hundreds of chemicals [51,52]. Traditionally, researchers have studied the chemosensory responses of *C. elegans* using nematode growth media (NGM) plates, using methods such as the drop test to observe behavioral changes in response to chemical stimuli. For example, Hillard et al. conducted drop tests in which a repellent was delivered near the tail of the animal using a glass micropipette to observe avoidance behavior [53]. However, these methods have limitations in delivering precise stimuli and performing neural functional imaging. To monitor the activity of ASH sensory neurons through calcium imaging, worms were immobilized using glue while chemical repellents and other stimuli were delivered. This immobilization method introduces uncertainties about its impact on actual neuronal activity.

**Figure 2:**
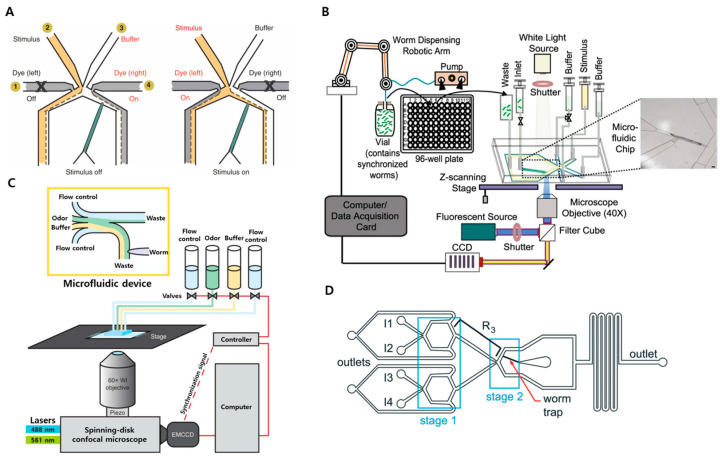
Examples of lab-on-a-chip applications for neural functional imaging of chemosensation in *C. elegans*. (**A**) The microfluidic device alternates between buffer and stimulus by switching two dye flows, enabling precise control of the chemical stimuli to worms. Reprinted with permission from [54]. (**B**) Diagram of an automated platform comprising a multi-well plate-to-microfluidics functional imaging module. Reprinted with permission from [38]. (**C**) Illustration of an automated platform consisting of a multi-well plate-to-microfluidic functional imaging system. Modified with permission from [39]. (**D**) Design of microfluidic device for rapidly switching chemical stimuli with a worm trap. Reprinted with permission from [40].

A breakthrough study introduced an olfactory chip that integrated a worm trap into a microfluidic chemical delivery system (Figure 2A) [36,37,54]. This design allows the trapping of a worm within the device channel without the need for glue or an anesthetic. By operating two-sided flows, the system can switch from stimulus to buffer streams, or vice versa. Another example is a fully automated platform comprising a multi-well plate-to-biochip interface module and a microfluid-coupled functional imaging module (Figure 2B) [38]. This setup enables the rapid evaluation of large-scale drug candidates through calcium imaging. Lin et al. designed a microfluidic chip with an arbor-containing delivery point for multiple stimuli, along with a buffer delivery point and two control switches: one for the buffer and one for the odor (Figure 2C) [39]. Rouse et al. have developed a platform capable of switching between multiple chemicals within milliseconds (Figure 2D) [40].

In addition to liquid-state chemical stimuli, chemosensation involves the detection of gas-state chemical stimuli. External gas signals, such as ambient oxygen, carbon dioxide, and various volatile odorants, provide animals with crucial environmental information [55]. Notably, *C. elegans* detects and adapts to these signals through its chemosensory system, aiding in the navigation of complex environmental changes. Conventional assays are conducted on plates to understand the mechanisms of gas sensation [56]. For example, carbon dioxide gas is pumped through a Pasteur pipette using a syringe pump and delivered near the head of a worm [57,58]. However, delivering gas to the desired stimulation site on NGM plates is challenging because of rapid gas diffusion during the delivery process.

**Figure 3:**
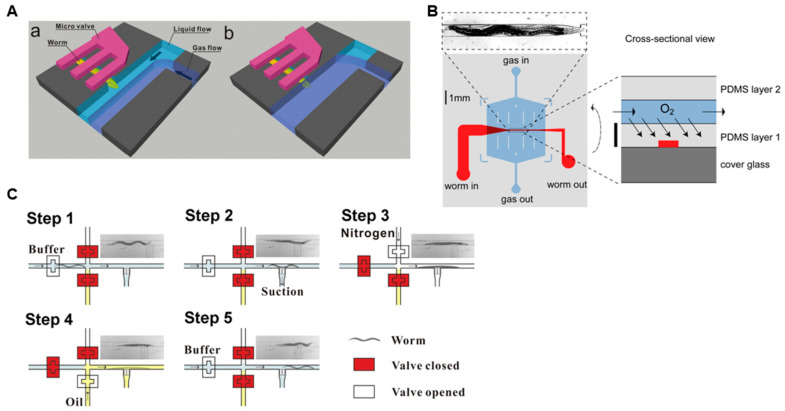
Microfluidic systems for investigating gas sensation in *C. elegans*. (**A**) Design of a microfluidic device consisting of a comb-shaped microvalve for worm immobilization and a T-junction structure for precise gas delivery. (**a**) Immobilized *C. elegans* was isolated from gas stimulus by buffer and N2 gas forming a two-phase laminar flow. (**b**) Gaseous stimulation applied through interface shifting. Reprinted with permission from [42]. (**B**) Two-layered microfluidic design featuring a worm channel (red) and an overlying O_2_ flow chamber (blue). The top image shows an animal in the channel. Reprinted with permission from [41]. (**C**) Microfluidic approach for immobilizing *C. elegans* by creating a gentle surface-dehydration condition. Gas stimulation is then applied for calcium imaging. Reprinted with permission from [44].

To overcome this limitation, several research labs have utilized lab-on-a-chip technology [41,42,43]. For example, Hu et al. implemented a microfluidic system using a comb-shaped microvalve activated by gas pressure for fixation, and a two-phase laminar flow to precisely switch gas stimulation (Figure 3A) [42]. Zimmer et al. developed a two-layered microfluidic device (Figure 3B) [41]. The bottom layer consisted of a worm-immobilization channel, whereas the upper layer contained a gas-flow channel bonded to the top of the worm channel. Due to the gas permeable polymer polydimethylsiloxane (PDMS), the approximately 75 μm PDMS partition between the two channels allowed rapid equilibration of O_2_ levels in the worm channel. This microfluidic design enabled brain-wide 3D imaging to establish the functional maps of neuronal networks [59,60].

Wang et al. injected nitrogen gas into a microfluidic chip to dehydrate the surface of *C. elegans* for rapid and repetitive immobilization (Figure 3C) [44]. This method is an alternative way to immobilize worms and allows gas stimulation without the need for a liquid-to-gas transition, enabling the investigation of the functions of gas-evoked neurons. These microfluidic-based systems enable the precise stimulation of specific worm parts with both liquids and gases, allowing for fixation and neural activity measurement without the use of anesthetics or adhesives.

### 3.2. Mechanosensation

Animals possess several senses that detect external mechanical stimuli, including touch, hearing, balance, and nociception [61]. Wild *C. elegans* live in nature and consume bacteria, relying on mechanoreceptor neurons (MRNs) to detect collisions with soil particles and bacterial food sources as well as forces generated by their own movement. Hermaphrodites and males have 22 putative MRNs, whereas males possess an additional 46 MRNs that are primarily involved in mating behavior [62]. Specifically, Chalfie and Sulston identified six touch receptor neurons (ALML/R, AVM, PLML/R, and PVM) necessary for gentle touch sensitivity, highlighting the simple and well-defined mechanosensory systems of *C. elegans* [63].

Conventional mechanosensation assays in *C. elegans* use manual methods. Glass probes with attached eyebrows or metal picks were used to apply gentle or rough stimuli to the anterior and posterior regions of *C. elegans* [64,65]. Additionally, pressure was applied to the worm, or the plate was tapped to induce behavioral responses [66]. These manual methods are difficult to control in terms of the stimulus intensity or the induction of consistent repetitive stimuli. Moreover, the animals are immobilized with glue to record neuronal responses using patch clamping or calcium imaging in response to precisely controlled mechanical stimulation. These methods limit the experimental throughput and prevent the recovery of animals for further experimentation.

**Figure 4:**
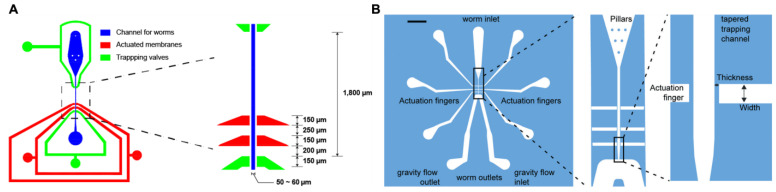
Microfluidic devices for neural functional imaging of mechanosensation in *C. elegans*. (**A**,**B**) Microfluidic device designs utilizing pneumatic PDMS actuators for the delivery of mechanical stimuli to specific regions of the worm body. (**A**) Reprinted with permission from [45]. (**B**) Reprinted with permission from [46].

To overcome these limitations, several microfluidic devices that deliver precise and reproducible mechanical stimuli have been developed. Some microfluidic devices utilize pneumatic PDMS actuators to apply mechanical stimuli to specific regions of *C. elegans* (Figure 4) [45,46,67]. These actuators can be finely controlled to deliver either gentle or harsh touch with high precision, allowing researchers to study mechanosensory responses in different parts of the body of the worm without the variability introduced by manual methods [45,46]. These devices feature an array of actuators that can be independently controlled to provide targeted mechanical stimuli, significantly reduce variability, and enhance the spatial and temporal resolutions. Cho et al. further refined this technology by miniaturizing pneumatic PDMS actuators for use with developing worms, such as the L2 larval stage, which enabled detailed studies of mechanosensory function development [47]. These advancements in microfluidic technology provide precise, reproducible, and high-throughput methods for delivering mechanical stimuli, overcoming the limitations of traditional manual methods and advancing the study of mechanosensation in *C. elegans*.

### 3.3. Thermosensation

All organisms, from bacteria to plants and animals, have mechanisms that react to environmental temperature changes that are crucial for survival, enabling them to respond to harmful temperatures or adapt to new environmental conditions [68]. Notably, *C. elegans* can not only survive and reproduce within a relatively broad temperature range of 12–26 °C, but also exhibit remarkable thermosensitivity, detecting and behaviorally responding to temperature changes as slight as 0.01 °C in laboratory settings [69,70]. In 1975, Hedgecock et al. identified thermostatic behavior in *C. elegans*, where worms migrated towards their preferred temperature in a temperature-gradient condition [71]. Mori et al. later identified AFD neurons as critical for thermosensation and thermotactic behavior in *C. elegans* [72].

In conventional methods, Peltier devices, which control and create temperature gradients, are widely used to monitor thermotaxis and measure neuronal responses from single to pan-neuronal levels [73,74]. However, these methods are typically suited for slow temperature changes, not acute stimuli. Heated aluminum tubes or infrared laser pulses have also been used to deliver temperature stimuli to worms [75,76]. Despite their effectiveness, these methods are not suitable for high-resolution functional imaging due to the limitations in their experimental design.

**Figure 5:**
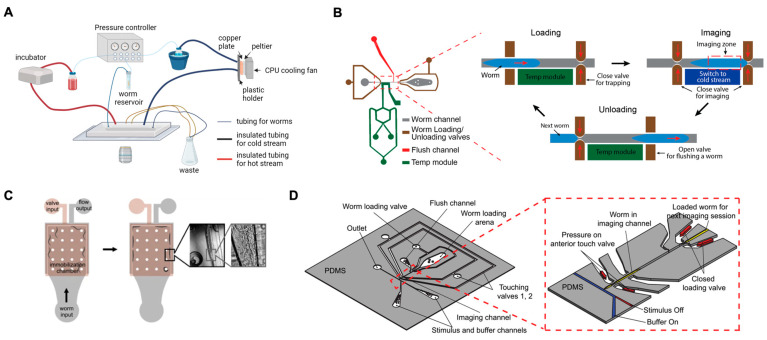
Advanced microfluidic applications for neural functional imaging in *C. elegans*. (**A**) Schematic illustration of a platform featuring a rapid flow-switching module for the delivery of acute cold shocks at the millisecond level. (**B**) Device design and automated operation procedures. (**A**,**B**) Reprinted with permission from [48]. (**C**) A two-layer microfluidic device with a valve layer above a flow layer, designed for precise immobilization of worms and the delivery of magnetic stimuli. Reprinted with permission from [49]. (**D**) A microfluidic device designed to deliver both mechanical and chemical stimuli through PDMS actuators and off-chip solenoid valves, respectively. Reprinted with permission from [50].

Microfluidic technology enables the precise and accurate delivery of temperature stimuli to *C. elegans* owing to several key advantages. The fine spatial control of microfluidic channels allows for localized heating or cooling in specific regions. The small volumes of these systems facilitate rapid thermal equilibration, enabling rapid temperature changes. The integrated sensors and actuators provide real-time feedback and control. Additionally, microfluidic systems support high-throughput processing of multiple samples using automated technology. Lee et al. developed a rapid flow-switching module integrating two temperature-control units, enabling the delivery of acute cold shock in milliseconds, while monitoring both the sensory and inter-neuronal responses of the worms (Figure 5A,B) [48]. This advancement highlights the potential of microfluidic devices in enhancing the study of thermosensation at neuronal levels in *C. elegans*.

### 3.4. Photosensation

Photosensation, which is the ability to detect and respond to light, is crucial for animals to navigate, avoid predators, find food, communicate, and regulate circadian rhythms [77,78]. Although *C. elegans* lacks eyes and specialized light-sensing organs, it can detect and respond to light. When a flash of blue, violet, or ultraviolet light is focused on the head of a forward-moving worm, it halts and initiates a reverse behavior [79]. The ASJ, ASK, AWB, and ASH neurons are critical for this head avoidance behavior, as their alteration results in a severe deficit in the response [80].

Traditional phototaxis assays involve placing worms at the center of an agar plate under light irradiation [80]. Light pulses are delivered to the head of the worm via the objective lens of a microscope, and the plate is manually moved to maintain the head of the worm in the field of view. However, this passive movement of the plate is imprecise, and as the worm continues to move, delivering light stimuli accurately to the desired location becomes challenging. To the best of our knowledge, no microfluidics-based system has been specifically designed to deliver light stimuli for functional imaging. However, confining worms within a microfluidic channel could potentially achieve precise delivery of light stimuli to specific locations on the worm. This approach would allow for a more accurate and reproducible investigation of photosensation in *C. elegans*.

### 3.5. Magnetosensation

Magnetosensation is a critical sensory process that enables animals to navigate, orient, avoid predators, and locate resources [81,82]. This ability is essential for the survival and reproductive success of many species, particularly those that migrate long distances or have complex navigational needs. Animals use magnetosensation as a compass to move towards favorable environments. Despite its significance, extensive research on the magnetosensory system has not been conducted in many animals.

Interestingly, even simple organisms, such as *C. elegans,* exhibit magnetosensation. Despite lacking complex sensory organs, *C. elegans* can detect and respond to magnetic fields, demonstrating the fundamental importance of this sensory ability across different species [83]. In *C. elegans*, the first pair of magnetosensory neurons (AFD) has been identified [84]. Traditional methods for studying magnetosensation often involve placing worms on NGM plates and applying external magnetic fields to observe their behavior. For example, magnetic fields can be created using an N42 Neodymium 3.5-cm diameter magnet [49]. Additionally, a 1 m^3^ volume tri-axial Merritt coil system can provide a uniform magnetic field similar to that of the Earth. The results showed that the movement and orientation of the worms were influenced by the presence of a magnetic field, with the worms preferring to move at specific angles relative to magnetic north. Although these conventional assays have provided valuable insights into magnetosensation, they are primarily designed as behavioral assays rather than neural functional imaging.

To directly observe the response of AFD neurons in *C. elegans* to magnetic fields, Vidal-Gadea et al. designed a microfluidic chip (Figure 5C) [49]. This device features a two-layer design with a valve layer above a flow layer where the worms reside. Worms enter the immobilization chamber, and fluid flow pushes them against the outer edge, where the valve layer pressurizes to fully immobilize worms, allowing for precise imaging and the delivery of magnetic stimuli using a magnet.

### 3.6. Multimodal Sensation

Environmental stimuli often trigger multiple sensory neurons that generate diverse sensory signals, which must be integrated and evaluated by the nervous system [85,86,87]. Signals from two or more sensory neurons are processed simultaneously to form a coherent representation of the environment. This integration is crucial for animals to effectively interpret complex environmental cues and enhance their ability to navigate, find food, avoid predators, and communicate. Notably, *C. elegans* is an ideal model system for studying multisensory integration in the nervous system because its connectome is fully mapped [88,89]. Despite its simplicity, *C. elegans* exhibits complex behaviors in response to a variety of sensory inputs, making it an excellent model for understanding the processes and integration of sensory information. However, due to the technical difficulties in developing technology for the delivery of multiple well-controlled stimuli to a worm, researchers have predominantly focused on single-stimulus experiments.

Cho et al. developed a microfluidic platform capable of delivering well-controlled mechanical and chemical stimuli with precise spatiotemporal and intensity patterns (Figure 5D) [50]. This system can deliver mechanical and chemical stimuli to *C. elegans* through deformable PDMS actuators and off-chip solenoid valves, respectively. Using this platform, Chew et al. and Cho et al. observed cross-modal sensitization at both the neuronal and behavioral levels [90]. The precise control and versatility of microfluidic systems offer significant potential for furthering our knowledge of neural processes, making them useful tools in the field of neurobiology.

## 4. Conclusions and Future Perspectives

The synergy between lab-on-a-chip technology and *C. elegans* as a model organism has not only advanced our understanding of basic neuroscience, but also promises high-throughput genetic screens and phenotypic assays. Microfluidic technologies have emerged as transformative tools that allow precise control of the microenvironment and delivery of stimuli. These devices facilitate high-quality neural functional imaging by confining *C. elegans* within microchannels, eliminating the need for drug-induced immobilization, and enabling easy recovery and longitudinal studies. The application of these advanced methods has enhanced the study of various sensory modalities in animals, including chemosensation, mechanosensation, thermosensation, photosensation, magnetosensation, and multimodal sensation. By enabling the precise manipulation and observation of neural activities, these technologies have provided deeper insights into how *C. elegans* and, by extension, humans perceive and respond to environmental cues.

As lab-on-a-chip technologies continue to evolve and merge with new neural imaging techniques, they are expected to drive significant advancements in the field. One promising direction is the development of more sophisticated, multi-functional lab-on-a-chip platforms that can simultaneously monitor multiple sensory modalities and their interactions. These platforms could incorporate advanced imaging techniques that offer higher resolution and better temporal dynamics, enabling the exploration of more complex neural circuits and behaviors in *C. elegans*. Such innovations would allow researchers to gain a more comprehensive understanding of sensory processing and integration.

Furthermore, recent advances in neuromorphic imaging, including the development of all-optical neural networks [91,92], multichannel imaging techniques [93,94], and neuromorphic chips [95], highlight the evolving landscape of neural functional imaging.

Additionally, the application of these technologies is likely to expand beyond *C. elegans* to other model organisms and even to ex vivo tissue systems. For instance, adapting lab-on-a-chip platforms for use with mammalian neurons or organoids could bridge the gap between invertebrate models and human neural systems, enabling translational research that is more directly applicable to human health.

The combination of high-throughput capabilities with automated data analysis tools, including machine learning algorithms, could also significantly enhance the scalability of these studies. This would facilitate large-scale genetic screens, drug testing, and the study of complex neural dynamics over extended periods, leading to discoveries that could inform the development of new treatments for neurological diseases.

In conclusion, ongoing innovations in lab-on-a-chip technology and neural functional imaging, combined with the robust capabilities of *C. elegans*, continue to provide a powerful platform for neuroscience research. The future of this field holds great promise, with potential applications that extend far beyond this simple nematode, impacting broader areas of neurobiology and biomedicine.

## Figures and Tables

**Table 1:** Summary of Lab-on-a-Chip applications for Neural Functional Imaging in *C. elegans*.

Modality	Principle	Target Neurons	Applications	Reference
Chemosensation	-Trap a worm in the channel -Switch flow using two-sided channels	ASH	-Automated microfluidic platform -Age-dependent ASH neuronal responses	Chronis et al., 2007 [36]; Chokshi et al., 2010 [37]
	-Utilize a multi-well plate-to-biochip interface and a microfluid-coupled imaging module.	ASH	-Measure lifespan-extending effects on ASH function -Screen FDA-approved compounds and validate targets	Bazopoulou et al., 2017 [38]
	-Immobilize and position a worm in the channel -Deliver pulses of single and mixed odorant solutions	11 pairs of amphid chemosensory neurons	-Simultaneously record 11 neuronal responses using volumetric imaging	Lin et al., 2023 [39]
	-Sequentially deliver multiple chemical cues at millisecond intervals using a pressure-driven chemical selector network	ASH, AWC	-Observe neuronal habituation -Monitor neuronal responses to higher stimulation frequencies with multiple chemical cues	Rouse et al., 2018 [40]
	-Employ a two-layer system for immobilization (bottom) and gas flow (top)	URX, BAG	-Identify URX and BAG neuron roles in O_2_ sensing -Investigate the involvement of soluble guanylate cyclases (sGCs) in O_2_ sensing	Zimmer et al., 2009 [41]
	-Fix worms using a gas-activated microvalve -Switch gas stimulation via a two-phase laminar flow.	URX, BAG, ASH	-Monitor neuronal responses to non-polar (O_2,_ CO_2_) and polar (1-octanol) gaseous stimuli	Hu et al., 2015. 2016 [42,43]
	-Inject nitrogen gas into a chip for rapid and repetitive immobilization by surface dehydration	URX	-Monitor URX activity relative to oxygen levels	Wang et al., 2017 [44]
Mechanosensation	-Use pneumatic PDMS actuators to deliver controlled mechanical stimuli -Automate imaging by streamlining worm handling	Six touch neurons, PVD	-Monitor habituated neuronal responses to the repeated mechanical stimuli -Examine the feasibility of drug screening based on neuronal activity using an orphan ligand library	Cho et al., 2017 [45]
	-Use pneumatic PDMS actuators to deliver controlled mechanical stimuli	Six touch neurons	-Monitor touch neuron responses to various stimulus patterns (e.g., step, ramp, and buzz stimulus)	Nekimken et al., 2017 [46]
	-Miniaturizing PDMS actuators for developing worms (L2)	Six touch neurons, PVD	- Investigate the effect of a sleep-like state on neuronal circuit activity to mechanical stimuli	Cho et al., 2018 [47]
Thermosensation	-Rapidly switch two difference temperature streams to deliver acute temperature stimuli (e.g., cold shock)	PVD, PVC	-Monitor sensory and inter-neuronal responses to cold shock stimulus -Observe habituated or sensitized neuronal response to repeated temperature stimuli	Lee et al., 2024 [48]
Magnetosensation	-Immobilize worms with a valve layer -Deliver magnetic stimuli using a magnet	AFD	-Observe AFD neuron responses to Earth-strength magnetic fields -Identify the role of the cGMP-gated ion channel TAX-4 in AFD magnetosensation	Vidal-Gadea et al., 2015 [49]
Multimodal Sensation	-Control mechanical and chemical stimuli with precise spatiotemporal and intensity patterns -Using pneumatic PDMS actuators for mechanical stimuli and flow switching for chemical stimuli	ASH, AVA, PVC	-Observe same and cross-modal sensitization at the neuronal level -Investigate the effects of Neuromodulators on PVC interneuron cross-modal Sensitization	Cho et al., 2020 [50]

## Data Availability

Not applicable.
